# Rapid non-genomic signalling by 17*β*-oestradiol through c-Src involves mTOR-dependent expression of HIF-1*α* in breast cancer cells

**DOI:** 10.1038/bjc.2011.349

**Published:** 2011-09-06

**Authors:** S Sudhagar, S Sathya, B S Lakshmi

**Affiliations:** 1 Centre for Biotechnology, Anna University, Chennai 600025, India

**Keywords:** oestradiol, HIF-1*α*, c-Src, mTOR, breast cancer

## Abstract

**Background:**

Hypoxia-inducible factor 1 (HIF1) has been implicated in regulating many of the genes responsible for angiogenesis, erythropoiesis, glucose metabolism and cancer pathogenesis. In this study, we demonstrate that exposure of human breast cancer lines to 17*β*-oestradiol (E2) rapidly induced the expression of HIF-1*α*, the regulated subunit of HIF1, in normoxic condition. Hypoxia-inducible factor-1*α* is normally degraded in normoxia through ubiquitination-mediated proteolysis, whereas hypoxia modulates HIF-1*α* level by inhibiting ubiquitination-mediated degradation.

**Methods:**

Oestradiol-induced accumulation of HIF-1*α* in breast cancer lines was detected by western blot analysis and its promoter activity was measured by HIF1 reporter assay. Molecular signalling of oestradiol-mediated HIF-1*α* expression was studied using specific pharmacological inhibitors and small interference RNA by co-immunoprecipitation and western blotting analysis.

**Results:**

Oestradiol has been observed to rapidly activate the nongenomic signalling cascade leading to HIF-1*α* protein synthesis. The results define a signalling pathway in breast cancer cells whereby oestradiol induces a rapid protein–protein interaction of ER*α*-c-Src-PI3K, resulting in the activation of PI3K/AKT pathway leading to mammalian target of rapamycin (mTOR) phosphorylation. The mTOR then stimulates translation by phosphorylating p70 S6 kinase and 4EB-P1, modulating HIF-1*α* protein synthesis. Oestradiol-stimulated HIF-1*α* activity was inhibited by either siRNA or pharmacological inhibitors to ER*α*, c-Src, PI3K and mTOR, providing a mechanism for the modulation of HIF-1*α* protein synthesis.

**Conclusion:**

These results show oestradiol-induced expression of HIF-1*α*, downstream of the ER*α*/c-Src/PI3K/AKT/mTOR pathway in human breast cancer cells.

The steroid hormone oestrogen exhibits pleiotropic effect on diverse physiological and pathological processes including breast cancer progression (Clemons and Goss, 2001). The role of oestrogen in the development and growth of breast cancer has been firmly established, although the precise molecular mechanism remains elusive. Oestrogen exerts its function through binding oestrogen receptor-*α*, a ligand-inducible transcription factor belonging to nuclear receptor superfamily and regulating gene expression by directly binding to oestrogen-responsive element (ERE) in the promoter region of DNA (Tsai and O'Malley, 1994). However, the transcription activity of oestrogen alone is not sufficient to fully explain its role in cancer development. Recent reports have indicated that oestrogen rapidly activates cytoplasmic signalling including c-Src kinase, phosphatidylinositol 3-kinase (PI3K)/protein kinase B (Akt) and the extracellular signal-regulated kinase (ERK 1/2) pathway (Haynes *et al*, 2003; Marino *et al*, 2003; Cabodi *et al*, 2004; Moriarty *et al*, 2006). However, the functional significance of non-genomic signalling of oestrogen in breast cancer has not been studied extensively. Dysregulation or activation of c-Src kinase and PI3K/Akt pathway are strongly associated with elevated level of hypoxia-inducible factor-1*α* (HIF-1*α*), a regulatory subunit of HIF1 expression in a variety of cell types (Jiang *et al*, 2001; Laughner *et al*, 2001; Treins *et al*, 2002; Arsham *et al*, 2002; Fukuda *et al*, 2002; Karni *et al*, 2002). The hypoxia-induced factor belonging to the family of transcription factor has been identified as an important regulator of cellular response to hypoxia.

Hypoxia-inducible factor 1, a basic helix-loop-helix transcription factor of PAS family, contains constitutively expressed HIF-1*β* and oxygen-sensitive HIF-1*α*. Hypoxia-inducible factor regulates the target gene by binding to hypoxia-responsible element (HRE) on the genomic DNA. Adaptation to hypoxia is vital for cancer progression, and cellular response to hypoxia is mediated through changes in gene expression primarily by hypoxia-inducible factors. The HIF1 transcription activity is primarily regulated through the stability of HIF-1*α* protein (Wang *et al*, 1995; Semenza, 2003). Elevated HIF-1*α* level is associated with the development of multiple neoplasms in Von Hippel–Lindu (VHL) disease and poor patient survival in breast cancer, signifying a decisive role in cancer development (Maxwell *et al*, 1999). Hypoxia-inducible factor-1*α* is scarce in normoxia, although it is constitutively transcribed, translated and rapidly degraded through hydroxylation at proline 402 and 564 residues by prolyl-4-hydroxylase (PHD), targeting HIF-1*α* for proteosomal degradation mediated by the VHL protein (Jaakkola *et al*, 2001).

Once stabilised, HIF-1*α* heterodimerises with HIF-1*β* subunit and recruits the co-activator, including CBP/P300 in the nucleus, and binds to the HRE in the promoter/enhancer to trigger the transcription of numerous hypoxia-inducible genes that promote cell survival, angiogenesis and glucose metabolism. It has also been associated with a variety of tumours and oncogenic pathways and has been a prime target for anticancer therapies (Freedman *et al*, 2002; Harris, 2002). Recently, oestrogen has been reported to induce HIF-1*α* and VEGF in the uterus of rats through the activation of PI3K/Akt, although the important mediators of the pathway remain elusive (Kazi and Koos, 2007). Given the role of HIF-1*α* in cancer progression and its activation by receptor tyrosine kinases (RTKs), the current study investigates the role of oestrogen in stimulating HIF-1*α* expression in breast cancer cells.

Here, we present evidence that 17*β*-oestradiol (E2), the most potent form of circulating oestrogen, stimulates steroid-starved ER*α-*positive breast cancer cells by rapid accumulation of HIF-1*α* and activates HIF1 through its non-genomic signalling. The E2-induced HIF-1*α* expression involves enhanced HIF-1*α* translation mediated by mammalian target of rapamycin (mTOR) signalling. These data support a model of E2-mediated HIF-1*α* expression through ER*α*/c-Src/PI3K/Akt/mTOR signalling pathway in breast cancer cells.

## Materials and methods

### Reagents and antibodies

All chemicals and inhibitors were purchased from Sigma-Aldrich (St Louis, MO, USA). All antibodies were obtained from Cell Signal Technologies (Danvers, MA, USA). Cell culture medium, fetal bovine serum (FBS), Lipofectamine 2000, RT–PCR reagents and enzymes were procured from Invitrogen (Carlsbad, CA, USA). Dual-Luciferase assay system was purchased from Promega (Madison, WI, USA).

### Cell culture and treatments

The cell lines MCF-7, T47D and MDA-MB-231 were obtained from ATCC (Manassas, VA, USA). The cell lines MCF-7 and MDA-MB-231 were grown in DMEM high glucose supplemented with 10% FBS, and T47D was grown in RPMI-1640 with 10% FBS. Prior to experiments, cells were made steroid starved for rapid oestrogen signalling studies by incubating them for 48 h in phenol red-free RPMI or DMEM in the presence of 5% charcoal-treated serum for respective cell lines.

### Cell extraction, immunoprecipitation and immunoblotting

Total cell extracts were prepared with the ELB extraction buffer: 50 mM Tris (pH 7.5), 0.1% NP-40, 120 mM NaCl, 1 mM EDTA, 6 mM EGTA, 20 mM NaF, 1 mM Na pyrophosphate, 30 mM 4-nitrophenyl phosphate, 1 mM benzamidine, 0.4 mM Na3VO4, 10 mg ml^–1^ leupeptin, 4 mg ml^–1^ pepstatin and 0.1 Unit ml^–1^ aprotinin. Cells were rinsed twice with ice-cold PBS and extracted on ice with ELB buffer. Typically, cell extracts were kept at −80 °C overnight and the soluble fractions cleared of debris the following day with a 14 000 r.p.m. spin for 25 min at 4 °C and assayed for protein concentration with standard Bradford protein assay method. A stock of the extract was made in Laemmli buffer and stored at −20 °C for western blot analysis. For co-immunoprecipitation experiments, 200 *μ*l of cell lysate was immunoprecipitated with antibodies to oestrogen receptor-*α* overnight at 4 °C followed by 1 h of incubation in the presence of 50 *μ*l protein A-Sepharose beads. Following SDS–PAGE, proteins were transferred to nitrocellulose, incubated with specific antibodies and then detected with either alkaline phosphatase- or peroxidase-conjugated secondary antibodies. When appropriate, the nitrocellulose membranes were stripped according to the manufacturer's recommendations and re-probed.

### siRNA transfection

All RNAi experiments were carried out with three pre-validated Mission siRNA directed against c-Src, Akt1/2 and mTOR from Sigma-Aldrich using Lipofectamine 2000 transfection reagent. For HIF1 promoter activity, cells were plated in 96-well plates, transfected with 20 nmol l^–1^ siRNA for 4 h, followed by 48 h in oestrogen-free medium. For western blot analysis, cells were seeded in 60 mm Petri dish and transfected with 80 nmol l^–1^ siRNA for 4 h, followed by incubation for 48 h.

### Plasmids and reporter assay

Cells were co-transfected with HRE luciferase plasmid containing three potent HRE sequences cloned into the pGL3 promoter luciferase reporter system and pRL-CMV vector as control using Lipofectamine 2000 as per the manufacturer's protocol. Transfected cells were steroid starved for 48 h and incubated with E2 (10 nM) at indicated time points, and Luciferase activity was measured and normalised relative to *renilla* luciferase units using dual Luciferase reporter system as per the manufacturer's instruction. Results are expressed as fold induction over control. Results shown represent the mean±s.d. of three independent experiments.

### RNA isolation and RT–PCR

Steroid-starved MCF-7, MDA-MB-231 and T47D cells were stimulated with 10 nM E2 at indicated time periods in six-well tissue culture plates. Total RNA was extracted using Trizol reagent and cDNA conversion was carried out using MMLV-Reverse Transcriptase as per the manufacturer's instruction. The RT–PCR was performed using oligonucleotide primer corresponding to *HIF-1α* cDNA and *GAPDH* as control. The PCR products were resolved in 1.8% agarose gel.

## Results

### Upregulation of HIF-1*α* and HIF1 activity in breast cancer lines by E2 stimulation

**Figure 1:**
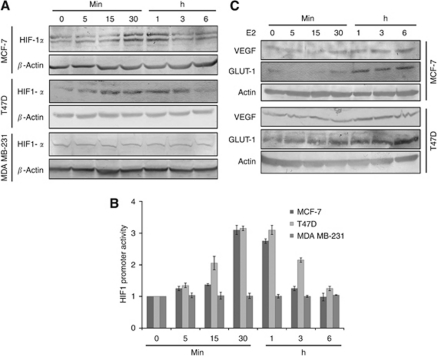
Hypoxia-inducible factor-1*α* (HIF-1*α*) protein level and HIF1 activity are upregulated in breast cancer cells by E2 stimulation. (**A**) Steroid-deprived MCF-7, T47D and MDA MB-231 cells were stimulated with E2 (10 nM) at the indicated time points. Whole-cell lysates were prepared and analysed by western blotting using antibodies to HIF-1*α*. Expression of HIF-1*α* was normalised to actin. (**B**) Breast cancer lines were co-transfected with HRE-pGL3 promoter luciferase reporter system and control vector encoding for the *Renilla* luciferase gene under the control of the CMV promoter. Transfected cells were steroid starved for 48 h and incubated with E2 (10 nM) at indicated time points and Luciferase activity was measured and normalised relative to *renilla* luciferase units using Dual Luciferase reporter system. Results shown represent the mean±s.d. of three independent experiments. (**C**) Expression of HIF1 downstream target genes was analysed by western blotting. Culture condition and E2 stimulation are similar to (**A**). The VEGF and GLUT-1 levels were normalised to actin.

Growth factor-stimulated RTK-induced HIF-1*α* expression has been reported in different cell types (Fukuda *et al*, 2002; Alam *et al*, 2009; Xu *et al*, 2010). To investigate the possible effect of E2 on the HIF-1*α* expression, steroid-starved ER*α*^+^ breast cancer lines MCF-7 and T47D and ER*α*^−^ MDA MB-231 cells were exposed to 10 nM E2, resulting in a time-dependent induction of HIF-1*α* protein expression. The expression of HIF-1*α* was stably detectable up to 1 h of exposure, after which a downregulation to basal level was observed. The E2-induced HIF-1*α* accumulation occurred only in ER*α*^+^ MCF-7 and T47D cells and not in ER*α*^−^ MDA MB-231 cells, potentiating the role of ER*α* (Figure 1A). To determine the effect of modulations in HIF-1*α* level on HIF1 transcription activity, the promoter activity of HIF1 using a HRE-pGL3 luciferase construct containing three potent hypoxia response elements relative to co-transfected pRL-CMV vector in breast cancer lines was measured. After treatment with E2 at different time periods, luciferase activity in cell extracts was determined and normalised to the *renilla* luciferase activity. Interestingly, HIF1 promoter activity was observed to be significantly increased in the breast cancer cells consistent with HIF-1*α* level on E2 stimuli. A three-fold increase in HIF1 transcription activity was observed after 30 min of E2 stimuli, which was maintained up to 1 h (Figure 1B). Investigation of the expression of known HIF1 target genes VEGF and GLUT-1 showed an upregulation in the protein expression after 1 h of E2 stimulation, exhibiting the significance of E2 modulation on HIF1 activity (Figure 1C).

### E2-mediated HIF-1*α* expression through translation-dependent pathway

**Figure 2:**
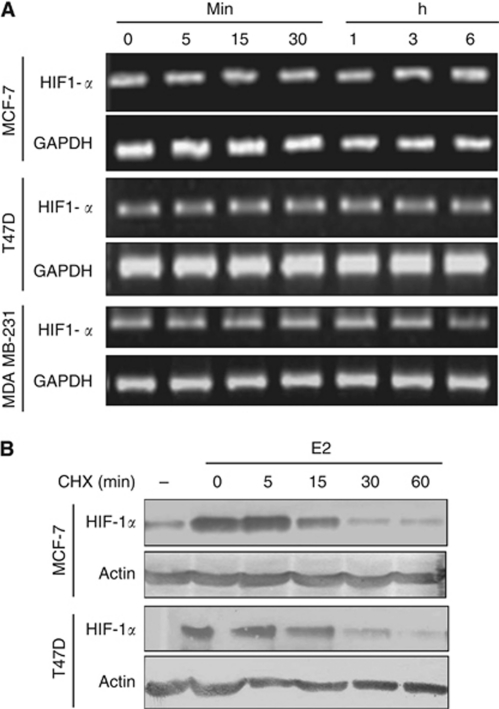
Through translation-dependent pathway, E2 mediated HIF-1*α* expression. (**A**) Steroid-starved cells were stimulated with 10 nM E2 at indicated time period in tissue culture plates. Total RNA was extracted and converted to cDNA. The RT–PCR was performed using oligonucleotide primer corresponding to *HIF-1α* cDNA and *GAPDH* as control. PCR products were resolved in 1.8% agarose gel. (**B**) The expression of HIF-1*α* was induced by exposure of 10 nM E2 for 30 min, followed by addition of cycloheximide (CHX), and MCF-7 and T47D cells were lysed after incubation at indicated time points. The cell lysates were resolved and analysed by western blot using HIF-1*α* or actin antibodies.

In general, oestrogen exerts its function through transcription, whereas regulation of HIF-1*α* occurs mostly at the protein level. Therefore, to understand the process responsible for HIF-1*α* accumulation in response to E2 treatment, the effect of E2 on *HIF-1α* mRNA expression in breast cancer lines was investigated. The expression of *HIF-1α* mRNA was observed to be similar at all the time points tested, suggesting that the elevated level of HIF-1*α* protein is independent of *HIF-1α* transcription (Figure 2A). As HIF-1*α* protein has been shown to be degraded in normoxia, we hypothesised that the elevated HIF-1*α* in response to E2 could be either through enhancing HIF-1*α* protein synthesis or modulating its stability. To pursue this hypothesis, a time course study of HIF-1*α* disappearance in the presence of protein translational inhibitor, cycloheximide (CHX), was performed. To this end, MCF-7 and T47D cells were treated with E2 for 30 min, followed by 100 *μ*M of CHX at different time points. The half-life of HIF-1*α* protein was observed to be markedly reduced after 5 min of CHX addition and was not detectable after 30 min (Figure 2B), thereby ruling out the possibility of E2 modulating the stability of HIF-1*α* protein. Together, our results suggest that E2 does not affect the HIF-1*α* stability and its mRNA transcription but instead dramatically increases the rate of HIF-1*α* protein synthesis.

### E2 rapidly activates non-genomic signalling of ER*α* through association with c-Src and p85-PI3K

**Figure 3:**
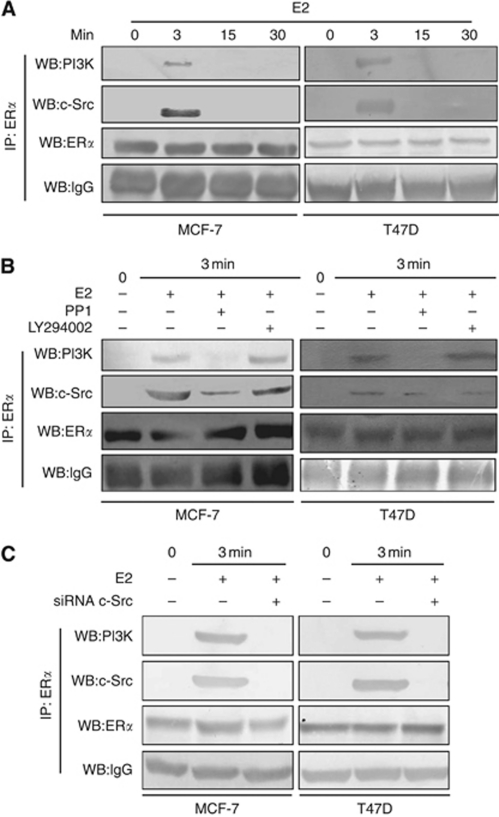
The ER*α*/c-Src/PI3K association is rapidly activated by E2. (**A**) Steroid-deprived MCF-7 and T47D cells were stimulated with E2 (10 nM) at indicated time points. Monolayer cells were washed, lysed and immunoprecipitated with anti-ER*α* antibody overnight at 4 °C and 1 h in the presence of 50 *μ*l protein A-Sepharose beads. The immunoprecipitates were immunoblotted with anti-c-Src antibody and re-probed with anti-PI3K (p85) antibody. (**B**) In parallel, steroid-deprived MCF-7 and T47D monolayers were pretreated with PP1 (10 *μ*M) and LY294002 (20 *μ*M) for 30 min and then stimulated with E2 for 3 min. Cells were washed, lysed and immunoprecipitated and immunoblotted with anti-c-Src and re-probed with anti-p85 PI3K antibody. (**C**) Cells were transfected with siRNA to c-Src and steroid starved for 48 h and then stimulated with E2 for 3 min. Cells were washed, lysed and immunoprecipitated and immunoblotted as mentioned above.

It has been known that oestrogen regulates intracellular signalling through interacting with non-RTK c-Src and the p85 subunit of PI3K. To assess the relevance of E2 signalling in the HIF-1*α* expression, steroid-starved breast cancer lines were stimulated with E2 at different time points. Cell lysates were co-immunoprecipitated with anti-ER*α* antibodies and analysed by immunoblot with either anti-p85 or anti-Src antibodies (Figure 3A). It was observed that E2 rapidly stimulates a transient macromolecular assembly between ER*α*, c-Src and PI3K at 3 min and this complex disappears at 15 min. In order to understand further the formation of macromolecular complex, co-immunoprecipitation was carried out in the presence of siRNA to c-Src as well as pharmacological inhibitors, PP1 and the PI3K inhibitor, LY294002.

Upon treatment with siRNA to Src and inhibitor PP1, PI3K was not detected in the immunoprecipitate of ER*α*, clearly showing that the inhibition of c-Src activity prevents the formation of the macromolecular complex, whereas LY294002 did not interfere with the ER*α*/c-Src/PI3K molecular assembly (Figure 3B and C). These results indicate the crucial role of c-Src in the formation of transient interaction between ER*α*/c-Src/PI3K as well as being the major upstream mediator of the non-genomic response of oestrogen in breast cancer lines.

### E2 activates mTOR signalling in breast cancer lines

**Figure 4:**
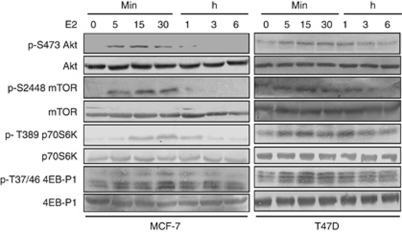
The Akt and mTOR signalling is activated by E2. The E2-dependent phosphorylation of Akt, mTOR, p70S6K and 4EB-P1. Steroid-starved MCF-7 and T47D cells were stimulated at different time points. The lysates were analysed by immunoblotting for the phosphorylation levels of Akt, mTOR, p70S6K and 4EB-P1 and its corresponding total protein level.

It has been well established that PI3K/Akt and mTOR pathway regulates HIF-1*α* expression through a variety of external stimuli. To elucidate the intracellular signalling pathway by which E2 mediates HIF-1*α* expression as well as HIF1 activation in breast cancer cells, the activation of downstream mediators linked to ER*α* signalling has been investigated. Akt is a classical downstream substrate of PI3K. As we and others demonstrate, E2-induced ER*α* rapidly interacts with the regulatory subunit of PI3K and this association strongly correlates with Akt phosphorylation. The transient macromolecular assembly induced by E2 between ER*α*/c-Src/PI3K was observed to accelerate activation of Akt phosphorylation in 5 min, which was found to disappear in a time-dependent manner (Figure 4). Several studies have suggested that Akt exerts its effect on HIF-1*α* by altering its translation through mTOR (Land and Tee, 2007; Li *et al*, 2007). Time course analysis clearly shows the phosphorylation of mTOR at 15 min of E2 induction. The phosphorylation of two important mTOR downstream effectors, p70S6K and 4E-BP1, was investigated and it was observed that E2 enhances the phosphorylation of p70S6K and 4E-BP1 from 15 min of E2 stimulation (Figure 4).

### PI3K/Akt and mTOR mediate the accumulation of HIF-1*α* in response to E2

**Figure 5:**
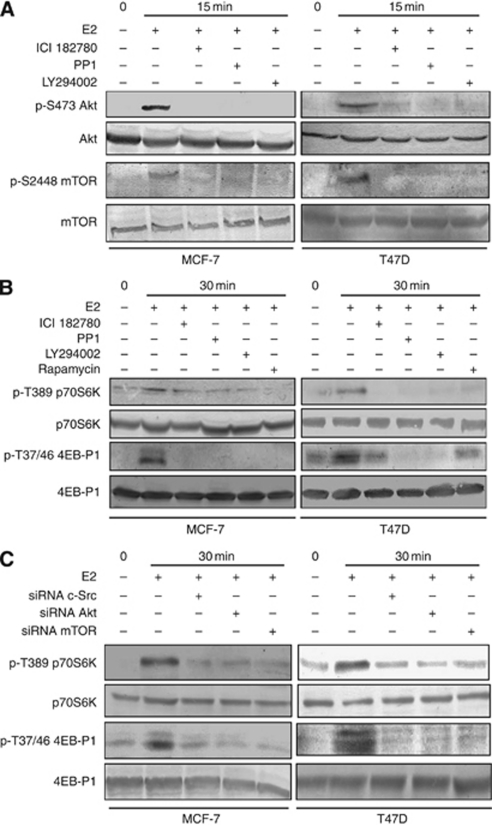
The E2 mediated signal through ER*α*, c-Src, PI3K and mTOR in breast cancer lines. (**A**) MCF-7 and T47D cells were cultured in steroid-free medium for 48 h. The ER*α* inhibitor ICI 182780 (10 *μ*M), c-Src inhibitor PP1 (10 *μ*M) and PI3K inhibitor LY294002 (20 *μ*M) were added for 30 min before the stimulation with 10 nM E2. The cells were incubated in the presence and absence of 10 nM E2 for 15 min. At the end of time point, cells were harvested and immunoblotting was used to detect the phosphorylation of Akt on Ser473, mTOR on Ser 2448 and the levels of Akt and mTOR. (**B**) Steroid-deprived MCF-7 and T47D cells were treated with ER*α*, c-Src, PI3K and mTOR (rapamycin 50 nM) inhibitors for 30 min, followed by 10 nM E2 for 30 min. Cells were lysed and immunoblotted with phosphorylated specific and total form of p70S6K and 4EB-P1 antibodies. (**C**) Cells were transfected with siRNA to c-Src, Akt and mTOR, followed by steroid starvation for 48 h. Cells were stimulated with E2 for 30 min and lysed. Cell lysates were subjected to immunoblotted with phosphorylated specific and total form of p70S6K and 4EB-P1 antibodies.

**Figure 6:**
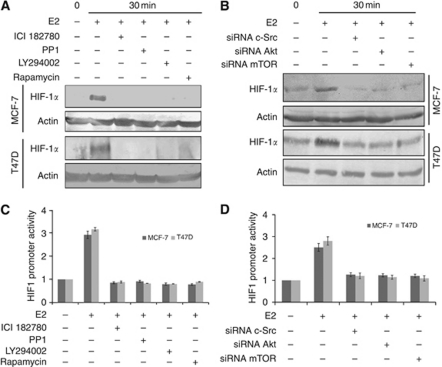
The ER*α*/c-Src/PI3K and mTOR activation mediates the accumulation of HIF-1*α* in response to E2. Pharmacological inhibitors or siRNA-mediated knockdown of ER*α*, c-Src, PI3k/Akt and mTOR affects HIF-1*α* expression induced by E2 stimulation. (**A** and **B**) Steroid-starved MCF-7 and T47D cells were incubated for 30 min either with ER*α* inhibitor ICI 182780 (10 *μ*M), c-Src inhibitor PP1 (10 *μ*M), PI3K inhibitor LY294002 (20 *μ*M) and mTOR inhibitor rapamycin (50 nM) or transfected with siRNA directed against c-Src, Akt and mTOR for 48 h, followed by 10 nM E2 stimuli. After 30 min of incubation, cells were lysed and immunoblotted with anti-HIF-1*α* and the blot was normalised with *β*-actin. (**C**) MCF-7 and T47D cells were co-transfected with HRE-PGL3 promoter plasmid and pRL-CMV vector for 24 h and the cells were treated with inhibitors of ER*α*, c-Src, PI3K and mTOR for 30 min and stimulated with 10 nM E2 for another 30 min and assayed for luciferase activity using Dual Luciferase assay system. (**D**) In all cases, MCF-7 and T47D cells were co-transfected with HRE-PGL3, pRL-CMV vector and with siRNA to c-Src, Akt and mTOR for 4 h, followed by steroid starvation for 48 h. Cells were stimulated with E2 for 30 min and assayed for luciferase activity using Dual Luciferase assay system. Results are expressed as fold induction of HIF1 activity normalised with *renilla* luciferase activity.

To demonstrate the E2-induced activation of ER*α*/c-Src/PI3K/Akt/mTOR signalling in HIF-1*α* accumulation, selective inhibitors or siRNA linked to E2 receptor and its downstream signalling pathway leading to HIF-1*α* translation were investigated. Our results clearly indicate that the pharmacological inhibitors of ER*α*, c-Src, PI3K and mTOR not only inhibited Akt/mTOR pathway (Figure 5A), but also inhibited the expression of HIF-1*α* (Figure 6A), confirming the importance of ER*α*/c-Src/PI3K assembly in the non-genomic signal transduction of E2 in breast cancer lines. Moreover, siRNA against c-Src, Akt and mTOR was observed to inhibit the phosphorylation of 4E-BP1 and p70S6K (Figure 5C) as well as exhibited blunted HIF-1*α* expression (Figure 6B) in response to E2, suggesting the importance of mTOR signalling in the elevated HIF-1*α* levels in breast cancer cells. To determine if these inhibitors block HIF1 promoter activity, the cells were transiently transfected with HRE-pGL3 and pRL-CMV vectors. Transfected cells were treated with E2 for 30 min in the presence and absence of inhibitors to ER*α*, c-Src, PI3K/Akt and mTOR. Cells treated with inhibitors of ER*α*, c-Src, PI3K and mTOR were observed to completely block HIF1 promoter activity, suggesting the importance of these signalling mediators in the activation of HIF-1*α* (Figure 6C). Similar results were observed using siRNA-mediated knockdown of c-Src, Akt and mTOR in the HIF1 promoter activity (Figure 6D). To summarise, the results clearly show that E2-induced HIF-1*α* level and HIF1 transcription activity was inhibited by pharmacological inhibitors, and siRNA directed to c-Src, PI3K/Akt and mTOR supports the view that E2-stimulated accumulation of the HIF-1*α* is strongly dependent on the ER*α*/c-Src/PI3K/Akt/mTOR pathway.

## Discussion

In this study, we show that oestrogen-induced HIF-1*α* accumulation in ER*α-*positive breast cancer leads to an activation of HIF1 in normoxic condition. Oestrogen rapidly activates the formation of transient macromolecular assembly between ER*α*/c-Src/PI3K, which further activates Akt and mTOR pathway promoting elevated levels of HIF-1*α* protein translation. The importance of HIF-1*α* as an oestrogen-inducible protein is of interest as it will enable us to understand further the oestrogen-induced cell proliferation and invasion in breast cancer. In breast cancer cells, HIF-1*α* is normally expressed at low levels in normal culture conditions and elevated during hypoxic milieu. The endogenous HIF-1*α* protein level is determined by the rate of protein translation and protein degradation. The degradation of HIF-1*α* is regulated by O_2_-dependent prolyl hydroxylation, which targets the protein for ubiquitylation by E3 ubiquitin-protein ligases (Jaakkola *et al*, 2001). Hypoxia induces HIF-1*α* accumulation in all cell types by inhibiting its ubiquitylated degradation pathway (Tanimoto *et al*, 2000). Interestingly, our results suggest that oestrogen does not affect the HIF-1*α* stability and its mRNA transcription but instead dramatically increases the rate of HIF-1*α* protein synthesis as shown by CHX chase and gene expression studies. Hence, we postulate that oestrogen enhances HIF-1*α* translation, leading to endogenous HIF-1*α* accumulation that could cause overwhelming ubiquitylation of HIF-1*α*, resulting in the degradation of only a subset of HIF-1*α* translated by oestrogen stimuli and independent of its stability. Taken together, our results strongly suggest that increased HIF-1*α* protein accumulation in breast cancer lines by oestrogen is largely dependent upon HIF-1*α* translation.

The role of oestrogen in breast cancer development has been studied mainly from the perspective of the transcription control of target genes via binding of its receptor to genomic consensus sequence (Clemons and Goss, 2001). Despite its classical mode of action, evidence has emerged that oestrogen could regulate intracellular signalling by interacting with non-RTK c-Src and the p85 subunit of PI3K (Castoria *et al*, 2001; Haynes *et al*, 2003; Cabodi *et al*, 2004). In our studies, upon treatment with oestrogen, ER*α* rapidly recruits a transient macromolecular complex with non-RTK c-Src and p85 subunit of PI3K. As we demonstrate in Figure 3B, ER*α* does not form a complex with PI3K in the presence of c-Src inhibitor PP1 or siRNA-mediated knockdown. We thus hypothesise that following oestrogen treatment, ER*α* binds to c-Src and allows the association of PI3K, revealing the importance of c-Src in oestrogen receptor non-genomic signalling. Reports have demonstrated that constitutively active c-Src expresses HIF-1*α* protein under normoxic condition, resulting in the activation of target genes. Our data have defined c-Src as a critical upstream activator in the oestrogen-stimulated HIF-1*α* expression in breast cancer lines. Pharmacological inhibitors or siRNA to c-Src not only inhibited Akt/mTOR activation, but also inhibited HIF-1*α* expression and its transcription activity, indicating that c-Src contributes to the dramatic expression of HIF-1*α* in response to oestrogen.

The accumulation of HIF-1*α* has been associated with cancer causing mutations including loss of VHL or PTEN and activation of signalling via RTK, non-RTK and G-protein-coupled receptor through activation of the PI3K and ERK MAPK pathways in a cell type-specific manner (Zundel *et al*, 2000; Zhong *et al*, 2000; Toschi *et al*, 2008). Moreover, reports have indicated that PI3K/Akt pathway occupies a central position in signalling, accepting inputs from diverse upstream sources like EGF, IGF and insulin in regulating the HIF-1*α* expression in normoxia and exhibiting limited effect under hypoxic condition (Zhong *et al*, 2000; Treins *et al*, 2002; Fukuda *et al*, 2002; Xu *et al*, 2010). Correlating with previous studies, our results also indicate that E2 rapidly activates Akt phosphorylation in serine 473 residue (Marino *et al*, 2003; Stirone *et al*, 2005). The macromolecular complex between ER*α*/c-Src/PI3K by E2 stimuli accelerates activation of Akt phosphorylation in 5 min that was inhibited by ICI 182780, PP1 and LY294002. Complete activation of Akt requires phosphorylation of at least two residues, Ser473 and Thr308. Activation of Akt correlates with the phosphorylation of highly inducible Ser473, whereas phosphorylation of Thr308 is thought to be majorly constitutive (Manning and Cantley, 2007). Our data support the conclusion that an elevated HIF-1*α* level and HIF1 activity was inhibited by treatment with PI3K inhibitor LY294002, and siRNA to Akt showed that oestrogen induced HIF-1*α* expression and HIF1 activity as a result of increased activity of PI3K/Akt pathway.

Several studies have suggested that Akt exerts its effect on HIF-1*α* by altering its translation through mTOR in normoxic condition, but is limited in hypoxic condition, where hypoxia suppresses the mTOR signalling pathway (Pore *et al*, 2006; Land and Tee, 2007; Li *et al*, 2007). The mTOR, a serine/threonine kinase, regulates cell survival and growth by integrating and transmitting signals from a diverse array of upstream activators by altering mRNA translation, ribosomal biosynthesis and metabolism (Ma and Blenis, 2009). The mTOR is a direct target of Akt and its activity has been regulated by direct phosphorylation of Ser2448 on mTOR correlating with its activation (Pore *et al*, 2006). The mTOR modulates the HIF-1*α* translation through cap-mediated mechanism that is driven by phosphorylating the translation repressor 4E-BP1, which inhibits 5′cap-dependent mRNA translation by binding and inactivating eIF4E (Land and Tee, 2007). Phosphorylation of 4E-BP1 leads to the release of eIF4E, allowing the formation of functional eIF4E complex and promoting the translation initiation of 5′cap mRNA (Richter and Sonenberg, 2005). The mTOR also regulates translation via P70S6K, which translates transcription encoding ribosomal proteins and translational elongation factors (Ma and Blenis, 2009). Hypoxia-inducible factor-1*α* has been reported to have a 5′cap structure as well as internal ribosomal entry site in its mRNA, allowing cap-dependent and cap-independent translations (Lang *et al*, 2002). Our results show that 4E-BP1 and P70S6K phosphorylation was inhibited by rapamycin or siRNA to mTOR and completely abolishes HIF-1*α* expression as well as HIF1 promoter activity in response to E2. This strongly suggests that mTOR signalling is downstream of PI3K/Akt pathway that regulates HIF-1*α* protein translation by oestrogen. Taken together, our results demonstrate the role of mTOR activation in the regulation of HIF-1*α* expression in response to oestrogen.

To conclude, ER*α*/c-Src/PI3K/Akt/mTOR signals elevate the levels of HIF-1*α* in response to oestrogen in ER*α*^+^ breast cancer lines. Oestrogen rapidly induces ER*α*/c-Src/PI3K macromolecular complex and places mTOR in control of HIF-1*α* translation. Also, E2 was observed to stimulate expression of HIF-1*α* through increased protein synthesis of HIF-1*α* without affecting its stability. The regulation of HIF-1*α* by E2 can explain, in part, the role of oestrogen signalling in breast cancer progression.
